# Leveraging open hardware to alleviate the burden of COVID-19 on global health systems

**DOI:** 10.1371/journal.pbio.3000730

**Published:** 2020-04-24

**Authors:** Andre Maia Chagas, Jennifer C. Molloy, Lucia L. Prieto-Godino, Tom Baden

**Affiliations:** 1 Sussex Neuroscience, School of Life Sciences, University of Sussex, Brighton, United Kingdom; 2 TReND in Africa, Brighton, United Kingdom; 3 Gathering for Open Science Hardware; 4 Department of Chemical Engineering and Biotechnology, University of Cambridge, Cambridge, United Kingdom; 5 The Francis Crick Institute, London, United Kingdom; 6 FENS-KAVLI Network of Excellence; 7 Institute for Ophthalmic Research, University of Tübingen, Tübingen, Germany

## Abstract

With the current rapid spread of COVID-19, global health systems are increasingly overburdened by the sheer number of people that need diagnosis, isolation and treatment. Shortcomings are evident across the board, from staffing, facilities for rapid and reliable testing to availability of hospital beds and key medical-grade equipment. The scale and breadth of the problem calls for an equally substantive response not only from frontline workers such as medical staff and scientists, but from skilled members of the public who have the time, facilities and knowledge to meaningfully contribute to a consolidated global response. Here, we summarise community-driven approaches based on Free and Open Source scientific and medical Hardware (FOSH) as well as personal protective equipment (PPE) currently being developed and deployed to support the global response for COVID-19 prevention, patient treatment and diagnostics.

## Abstract 2

In light of the Covid-19 pandemic, this Community Page article discusses the projects, pitfalls and possibilities regarding open source personal protective equipment, ventilators and diagnostics.

## Introduction

In recent days and weeks, governments around the world have called upon industry to address key shortcomings in the global response to COVID-19—for example to produce more personal protective equipment (PPE), ventilators and diagnostic tools [1,2]. While this is an important part of any country’s response, the capacity of existing industry to meet the scale of the challenge is likely insufficient [3–5]. Moreover, this approach will first address shortages locally where relevant industry is based, rather than globally, and medical grade equipment and kits emerging from this process will be costly, at a time where the economy is taking a big hit. Clearly, product innovation and novel manufacturing pipelines are required.

Here, one complementary access route to much needed tools and equipment is Free and Open Source (scientific and medical) Hardware (FOSH). FOSH follows the ethos of open source software, where all blueprints for a tool are made freely available under appropriate licenses so that anyone can study, learn, modify, customize and commercialize them [6,7]. Studies and practical experience with FOSH have shown key benefits that are paramount for disaster situations: fast and distributed development based on the contributions of many people who are for the most part working remotely [8,9].This is advantageous given the social distancing measures in place in many COVID-19 affected countries. The typically much lower implementation costs of FOSH [10] and easy adaptability to local resources are key further benefits of an open hardware approach.

Finally, and perhaps most importantly, any new hardware designs or improvements thereof are by their very definition globally available. Anyone equipped with the necessary knowhow, tools and time can build on this knowledge to meaningfully support their immediate community. The importance of the latter cannot be overstated: Different communities face different limitations in the availability of trained staff, medical consumables and machines as well as diagnostic tools. Accordingly, what may be limiting in one place may not be limiting in the next, and any global response must therefore be adjusted to local realities. Here, the many benefits of a FOSH approach allow for fast, local deployment which can bypass traditional production chains to flexibly supply affected areas as they emerge. While this is useful worldwide, it may be particularly important for regions that traditionally have fewer communication links and/or where the medical and scientific infrastructure is generally less well developed [11].

## A FOSH approach to supporting global health systems

Increasingly over recent years, scientists, engineers and hobbyists alike have jointly developed and tested an impressive array of open source and state-of-the art tools that in one way or another touch all aspects of modern biology, medicine and disaster response (e.g., [12–23]). For example, in the wake of the 2011 Fukushima nuclear disaster, Safecast [9] developed FOSH Geiger counters alongside an open access logging system which led to a massive citizen-science driven map of nuclear contamination in the region [9]. Now the same group is stepping up to meet the challenges of COVID-19 [24]. Other community driven FOSH designs relevant to the current situation range from simple tools like DIY masks [25,26] or 3D printed valves to regulate airflow in ventilator tubes [27] to state-of-the-art scientific instruments for diagnosis such as an automated pipetting robot [28], plate readers [29] as well as a wide range of medical tools and supplies [22]. Diverse further initiatives, including numerous designs for FOSH ventilators [30–38], are well underway. Here, we provide a brief overview of the current state-of-the art in available designs and ongoing community projects aiming to leverage FOSH to meaningfully contribute to a global response to the current crisis (see also Box 1). In the specific background of COVID-19, we highlight a subset of available projects centered around:

1. Personal protective equipment (PPE) such as masks and visors
2. Patient treatment, focussing on ventilators
3. Diagnosis tools, focussing on scientific equipment and test-kits

Although several projects are listed here, interested readers can find curated lists online that are being updated on a daily basis. At the time of writing these were available [36,39,40]

**Box 1. An important note of caution and disclaimer**

**An important note of caution:** When implementing any of the projects mentioned in this article, it is important to critically evaluate the reliability and safety of the design on a case by case basis. While some designs have been extensively tested and verified, others are at best anecdotally verified and are intended for research use only. While this is important in all aspects of FOSH, it becomes imperative when considering diagnostic and medical tools. This should also include careful reference to regulations on control softwares, where applicable [41]. Use of these designs in a clinical setting may be prohibited by local regulations. Notably, the US National Institutes of Health (NIH) 3D print exchange collects a list of designs that have been approved for clinical use in at least one hospital [42].

**Disclaimer**: The introductory notes on masks and ventilators are based on available literature. The authors have no formal medical training.

## Building personal protective equipment (PPE)

**Fig 1:**
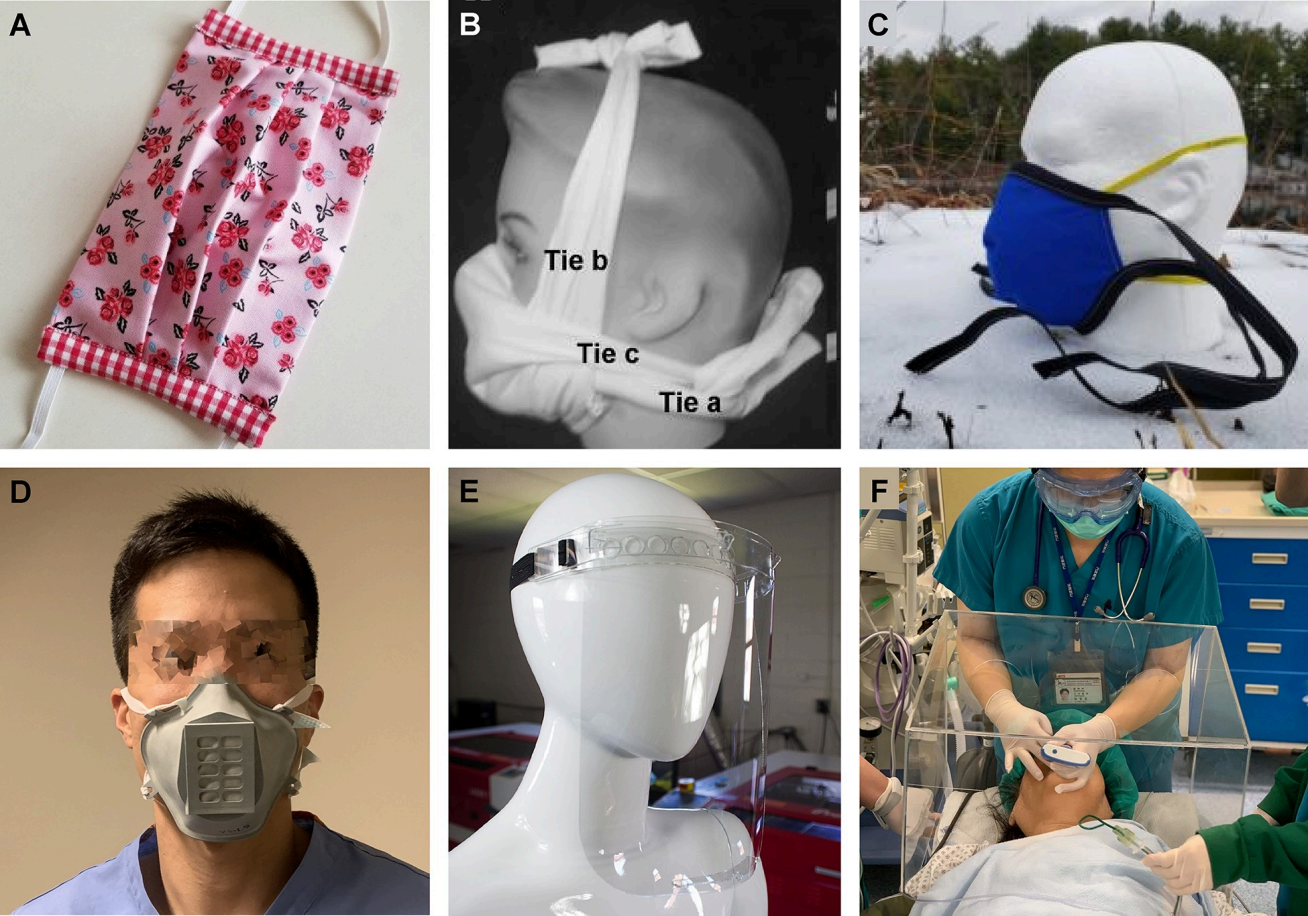
Examples of DIY masks and visors. **A**, One of many designs for a home-made cloth-mask [43], which to date remain generally untested for effectiveness. **B**, Somewhat more effective cotton T-shirt based mask [25], **C**, Impermeable mask cover [44]. **D**, Stop gap surgical mask (3D printed) [45]. **E**, Laser-cut face-shield [46]. **F**, Aerosol box [47].

Buying a facemask off the shelf is becoming increasingly difficult, and people are understandably looking into do-it-yourself (DIY) options that may serve as a useful replacement. Similarly, hospitals are running out of specialised personal protective equipment (PPE) for medical staff, which typically includes both a mask and a visor, alongside specialised clothing and gloves. Here, we will focus on DIY masks and visors (Fig 1, Table 1).

**Table 1:** Open source and do-it-yourself personal protective equipment. Highlighted are masks, face shields and aerosol boxes.

Project Name	License	Type	Note
**A, Surgical-type masks**			
Simple respiratory mask	N/A	Tied	Peer reviewed open access paper
3D printed face mask from la factoria 3d	CC-BY-NC (?)	3D printed	license from https://www.thingiverse.com/thing:4225667
Stopgap surgical face mask	CC-BY	3D printed	3D printed mask that has undergone clinical testing
Montana Mask	N/A	3D printed	Shares more than stl files for 3D printings
EDAGmask4all	N/A	3D printed	Mask clips to hold textile in place. Only stls are available
Bra face mask	N/A	Sewn	Masks made out of bras.
CDC face masks	N/A	Sewn	Cloth masks, CDC tutorial
Cloth mask with filter	N/A	Sewn	Cloth masks, Georgia Tech tutorials
DIY 2 layer cloth mask	N/A	Sewn	Cloth mask with 2 layers
DIY sewn facemask	N/A	Sewn	Cloth mask
How to sew a face mask	N/A	Sewn	Video tutorial
Maker Masks	CC BY 4.0	Sewn	Multiple designs including a mask protector
Paper towel mask	CC BY 3.0	Paper towel	Paper towel mask
**B, N95-type masks**			
Project 1000X1000	CC-BY-NC 4.0	Filter production	Initiative to produce air filters for masks License limits legal use in several places
Copper 3D	CC-BY-NC 4.0	3D printed	License limits legal use in several places Only sharing STL files
N95decon	Copyright	Test/study consortium	"A scientific consortium for data-driven study of N95 filtering facepiece respirator decontamination"
Pneumask	CC-BY-NC-SA 4.0	Snorkel mask	License limits legal use in several places
**C, Face protectors**			
Origami Droplet Face-Shield	CC-BY-SA 4.0	Laser cut	Shield using only laser cut parts
Proto-shield	CC-BY-SA	Laser cut	Shield using only laser cut parts
GliaX face protector	GNU-GPL 3.0	3D printed + sheet	
Prusa face protector	CC-BY-NC 4.0	3D printed + sheet	License limits legal use in several places
Georgia Tech face shields	N/A	Injection mold	
Face protector w/o 3D print/laser cut	N/A	Glued	
U. Wisconsin-Madison face shield	N/A	Glued	
Face protector from water bottle	N/A	Cut-out	
Prusa face protector disinfection tests	Copyright	Disinfection tests	Copyright is for webpage. N/A about project license
**D, Other PPE**			
Aerosol box	CC-BY-NC 4.0		License limits legal use in several places.
Aerosol container	CC-BY-NC 4.0		Based on aerosol box listed above
Barrier enclosure for endotracheal intubation	Copyright		

First, it is important to consider what level of protection is required. For example, masks come in many varieties, with different purposes and regulatory standards [48–51]. Some simpler masks (often called surgical masks) are intended to be used by the infected person to reduce spread of infection by catching large droplets, for example after coughing and sneezing. Such masks are fairly easily home-fashioned (e.g. [52], Table 1A), but they are not generally thought of as an effective form of protection from becoming infected in the first place [21,22,35]. Nonetheless, it has been argued that they can reduce the spread of viral particles from infected persons [53]. A recent influential paper [54] elaborated on this point, however this study did not look at SARS-CoV-2, nor at possible spread from asymptomatic carriers (see also [55]). Another study, with four COVID-19 patients, found surgical masks to be ineffective in preventing the spread of SARS-CoV2 [56]. Whether or not the general public should be advised to use surgical-type masks, and under which specific circumstances, is a matter of ongoing debate. At time of writing, the World Health Organisation (WHO) advises:

- If you are healthy, you only need to wear a mask if you are taking care of a person with suspected 2019-nCoV infection.
- Wear a mask if you are coughing or sneezing.

For an up-to-date summary on the role and use of various types of masks, the reader is referred to their official advice [57]. Notably, the Centre for Disease Control (CDC) in the USA currently recommends wearing masks in public settings where other social distancing measures are difficult to maintain, especially in areas of significant community-based transmission [58]. Moreover, it is possible, and arguably likely, that others will follow suit, especially as the world reopens.

In contrast to surgical masks, so called N95 filtering facepiece respirators (FFRs) are intended to provide a high level of protection to the wearer, and they are for example used by medical staff tending to infected patients. Unlike surgical masks, these are designed to seal against air slipping through gaps between the skin and the mask, and they contain a specialised filter that acts as a physical barrier to particles down to 0.3 microns. This is still bigger than the actual virus (~0.12 microns) [59], but substantially smaller than most droplets coming off a sneeze or cough. The performance standards for such masks are tightly regulated [48–51], and any DIY approach to replicating them must take careful reference to these regulations, which may also vary locally. Accordingly, controlled testing of existing and upcoming N95-like FFR prototypes is paramount. Moreover, unless worn correctly they may end up being no more useful than a surgical mask [57]. For protecting the eyes, N95s can be combined with tight-fitting goggles, or a face shield.

Right now there are many DIY designs for various types of masks out there, from simple video tutorials based on napkins, cloth or bra-cups (Table 1A) [60–62] via 3D printed options [63] to designs developed and/or tested in peer-reviewed scientific studies [25,26] (Fig 1). Some are quite effective [25], however to our knowledge to date none fully comply with regulatory standards. Nonetheless, with designs currently being published on a daily basis, this may soon change. Table 1B summarises a subset of DIY N95 projects available at time of writing.

Alternatively, medical staff also use splash-resistant surgical masks (IIR, resistant at 160 mmHg), together with a face shield [42]. The latter combination may be easier to build to regulatory standards than N95 masks [56], in part because they do not need to seal tightly against the skin. An online search quickly reveals several designs of face shields (and IIRs [44]) with varying levels of associated testing [64,65] (Table 1C, Fig 1E). For example, 3D Crowd is a UK-based citizen network that is mobilizing volunteers to produce and deliver DIY face protectors to hospitals and health institutions [66]. Through “The Big Print” they have gathered requests for 370,000 units. 80,000 of them are in production by currently over 5,500 volunteers. An alternative approach is based on existing full-face snorkel masks [67] which would effectively serve as a combined mask and visor. Efforts such as these, including their important experimental verification, could easily be scaled with minimal investment. They may also be more easily disinfected for reuse compared to masks [68,69].

In addition, more specialised PPE is also being actively developed. This includes DIY testing booths for diagnostics (virus inactivation before testing needs to be done in a biosafety level 2 facility, BSL2, see below) and DIY aerosol boxes, which are for example used when intubating patients (Fig 1F, Table 1D). Like all medical equipment, these also require regulatory approval [48–51].

## Choice of building materials for PPE

Building materials should be carefully considered. For example, it has been argued that the porosity of 3D printed materials could make them a risky choice for masks as they may allow viral droplets to persist for prolonged periods of time [68]. Possible gaps in the material itself (e.g. due to blistering or imperfect layering during printing) add further room for concern. At the time of writing, we are not aware of available data on the above points. Similarly, when laser cutting parts the use of acrylics should be carefully weighed against the need to sanitize it using alcohols or oxygen peroxide which can react with the acrylic. If available, and if the laser cutter at hand can handle it (many cannot), polycarbonate or polyethylene terephthalate glycol (PETG) sheets may present a safer choice. A guide to disinfecting DIY PPE can be found here [70]. Throughout, it also remains important to consider possible quality variations due to the building process itself.

## FOSH Ventilators

Ventilators are medical devices that support or take over patient respiration by delivering air or other gas mixtures. Patients able to breathe on their own are often treated supported by a constant airflow through cannulas or positive pressure masks (PPMs). In more severe cases patients are intubated (insertion of a tube into the patient’s airway). Here, some modern ventilators can synchronise the release of gases to any remaining natural breathing attempts. In other cases, ventilators can take over entirely (usually combined with patient sedation). Towards the end of treatment, patients must be carefully weaned off the ventilator. Generally, the use of ventilators brings about risks for infections including pneumonia, lung damage and oxygen toxicity. The latter two of which can sometimes be linked to improper use of the device (e.g. excess air pressure and/or oxygen concentration).

From a technical point of view, ventilators can be as simple as manually operated compressible bags (bag valve masks—BVM), or as complex as fully computerized machines that regulate gas pressure, humidity, gas relative concentrations as well as cycle rate, while taking real-time measurements to monitor patient condition. This added complexity of some more modern systems makes them more suitable for prolonged use, as they better mimic physiological conditions and can be adjusted to specific patient needs. The NIH provides a general overview on ventilators and their use [71], including a wide range of interface types, ventilator functions and other details that are not covered in this article.The UK also recently published their own specifications for ventilators to be used in hospitals [72].

The use of non-invasive systems for patients with COVID-19 in hospitals is currently debated due to their potential to create aerosols which increase the risk of infecting others [73]. Nonetheless some have argued they may have a place under certain circumstances [74], and it is moreover possible that such systems may come into increased use as the situation worsens. However, only invasive systems can fully take over respiration where required.

**Fig 2:**
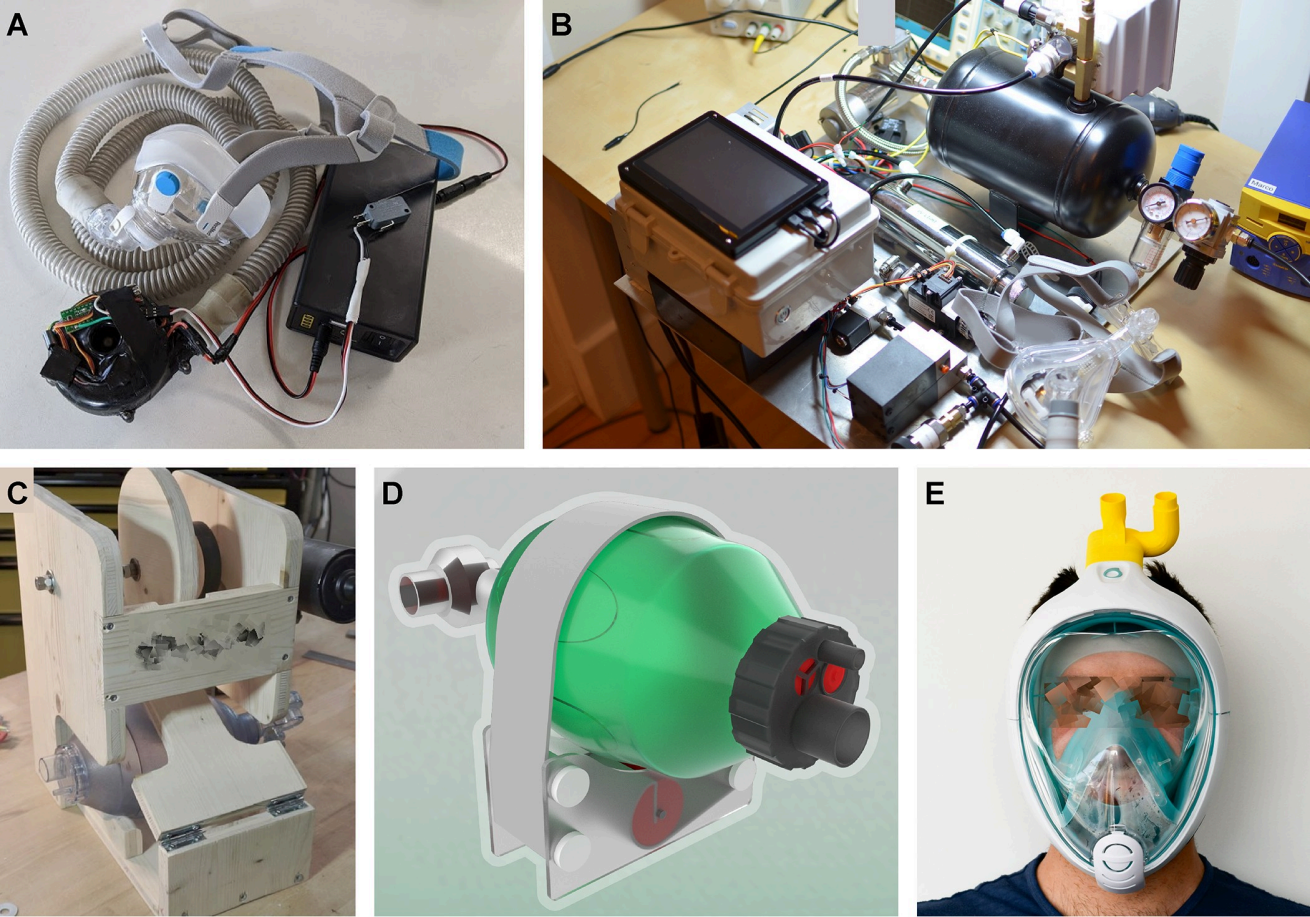
Examples of FOSH ventilators. **A**, Arduino based PPM system [31]. **B**, Pandemic ventilator derivative [84]. **C**, OxyGEN bag automation system [37], **D**, Prototype rendering from Open Source Ventilator initiative [85]. **E**, PPM ventilator based on snorkel mask and 3D printed adaptor [86].

Already now, and likely more so in the near future, availability of ventilators for patient care is limiting [75–77]. Current efforts to address this impasse include the stopping of elective surgeries to free up existing systems [78], the recommissioning of disused models from storage, and a plea to industry to ramp up production [1,79]. In parallel, diverse groups around the world are rallying their communities to design FOSH-solutions that will help increase the availability of ventilators (Fig 2, Table 2). Some examples include the development of complete and stand-alone systems [80,81], the automation of manual ventilators [31,33,37] and the repair of existing but out-of-use equipment, e.g. with 3D printed replacement parts [82,83]. Many projects are actively looking for collaborators with various backgrounds. FOSH ventilators for invasive use will need to implement feedback systems based on sensors that measure pressure, CO_2_ and oxygen levels, tidal and dead space volumes, and air humidity. These are necessary measurements to know if the machine is doing what is supposed to, and that it is not causing even more harm to the patient.

**Table 2:** Open source ventilators and positive pressure masks.

Project Name	License	Type	Note
Ventilators			
Open ventilator system initiative	CERN-OHL v2.0	Ventilator	Documentation to be released
Reesistencia	GNU-GPL 3.0	Ventilator	Advanced testing stage, updates regularly: https://twitter.com/ReesistenciaT?s=09
Inspire-OpenLung	CERN-OHL v2.0	Ventilator	Brazilian project, most documentation in Portuguese
CITI-OpenLung	CERN-OHL v2.0	Ventilator	Brazilian project, fork of "Inspire" above
Electrónica Reespirator23-17	MIT / CC-BY-NC 4.0	Ventilator	Repository is licensed with MIT, but design files are CC-BY-NC 4.0
Open Vent	Permissive coypright (?)	Ventilator	Started as part of the WirvsVirus hackathon
MUR	GNU GPL 3.0	Ventilator	Project in French
E-Vent	Not released yet	Ventilator	From MIT. No designs have yet been published.
Open Ventilator	N/A	Ventilator	
Open Source Ventilator Ireland	GNU-GPL 3.0	Ventilator	
Open Lung	GNU-GPL 3.0	Ventilator	Canadian project working with Irish Open Source Ventilator above
Take a breather (TAB)	N/A	Ventilator	
The Pandemic Ventilator	N/A	Ventilator	One of oldest projects, before "reactivation" posts date back to 2011
Pandemic ventilator 2.0	N/A	Ventilator	Pandemic ventilator spin off
Proyecto PVPv1.2	N/A	Ventilator	Pandemic ventilator spin off, based in Argentina
Oxygen	N/A	Ventilator	Spanish project with two versions: one for makers, one for industry
RespiraWorks	N/A	Ventilator	Project to document ventilators designs
Ventilator firmware	MIT	Ventilator	Brazilian project developing a universal firmware for ventilators
Medtronic ventilator	Custom license	Ventilator	License only allows use while WHO PHEIC is in place or Oct 2024
Oxvent	N/A	Ventilator	No designs available yet. Ventilator in testing phase in the UK.
Vent4us	N/A	Ventilator	Documentation to be released
PAPR	MIT	PPM Ventilator	
Mechanical ventilator Milano	N/A	PPM Ventilator	
Illinois rapid vent	Custom license	PPM Ventilator	Requires signature of license form
Isinnova Easy covid 19	N/A	PPM Ventilator	Positive pressure mask (PPM)
Ifixit repair guides	CC	Ventilator repair guide	

## FOSH approaches in COVID-19 diagnostics

The WHO recommends that countries should prioritize case finding, testing and isolation as a main strategy to slow down the spread of the virus [87]. This means that testing must be made widely available. The diagnosis of SARS-CoV-2 involves sample collection, usually from nasopharyngeal or oropharyngeal swabs [87], followed by RNA extraction as SARS-CoV-2 is an RNA virus. This typically involves binding the RNA onto silica in specific buffers followed by washing and resuspension. Then, the viral RNA is specifically amplified after reverse transcribing it to DNA. Finally the presence of this DNA is detected, usually by measuring changes in fluorescence intensity or colour. The most common diagnostic procedure involves the use of a commercial kit for RNA extraction, followed by quantitative Reverse Transcription Polymerase Chain Reaction (qRT-PCR) [88] which requires several types of hardware, chemicals and reagents to be deployed and typically takes place in a regulated lab that meets ISO 15189 standards for diagnostics [89]. In addition, RNA extraction or virus inactivation before RNA extraction elsewhere must take place in a BSL2 facility (see above for FOSH PPE designed to substitute a BSL2 facility).

Central testing labs overwhelmingly use automated sample extraction systems and other hardware that have been validated by the FDA and CDC in the US or the national equivalent in their local jurisdiction. These are supplied by a small number of large companies (e.g. Qiagen, Roche and Abbott) with vertically integrated business models: only proprietary reagent cartridges and plastic pipette tips for processing samples will typically fit the instruments and there are no or few generic suppliers. This is one of the reasons for acute shortages that have led to calls for donations of reagents and instruments from academic labs to bolster public health efforts [105].

There has also been unprecedented rapid and open sharing of diagnostics and devices through preprint publications, and through informal communication routes among researchers [90,106–108]. For example, agencies in China, Germany, Hong Kong, Japan, Thailand, South Africa and the United States have shared protocols for molecular assays online, including the DNA sequences to detect the virus. Others are now reproducing these in their own labs as well as commercially [88,109]. Meanwhile, large sums of money are being invested in universities, public hospitals and the private sector to develop rapid point-of-care (POC) or near-POC devices to enable scaling of testing in clinics, temporary facilities and even homes.

All reagents and devices used for diagnostics are typically regulated in terms of their manufacture and use, meaning that “home-brew” or DIY solutions are typically not acceptable unless the lab developing and using them is itself certified, and then only under certain conditions. In some jurisdictions regulations are becoming simpler or accelerated during the COVID-19 emergency and novel tests are starting to get approval for diagnostic use through the FDA’s Emergency Use Authorisation (EUA) scheme [68], WHO’s Emergency Use Listing and other national schemes [110]. The transparent and international sharing of protocols at inter-agency level is capable of driving forward testing while academic and commercial projects catch up and apply for emergency approval status.

**Fig 3:**
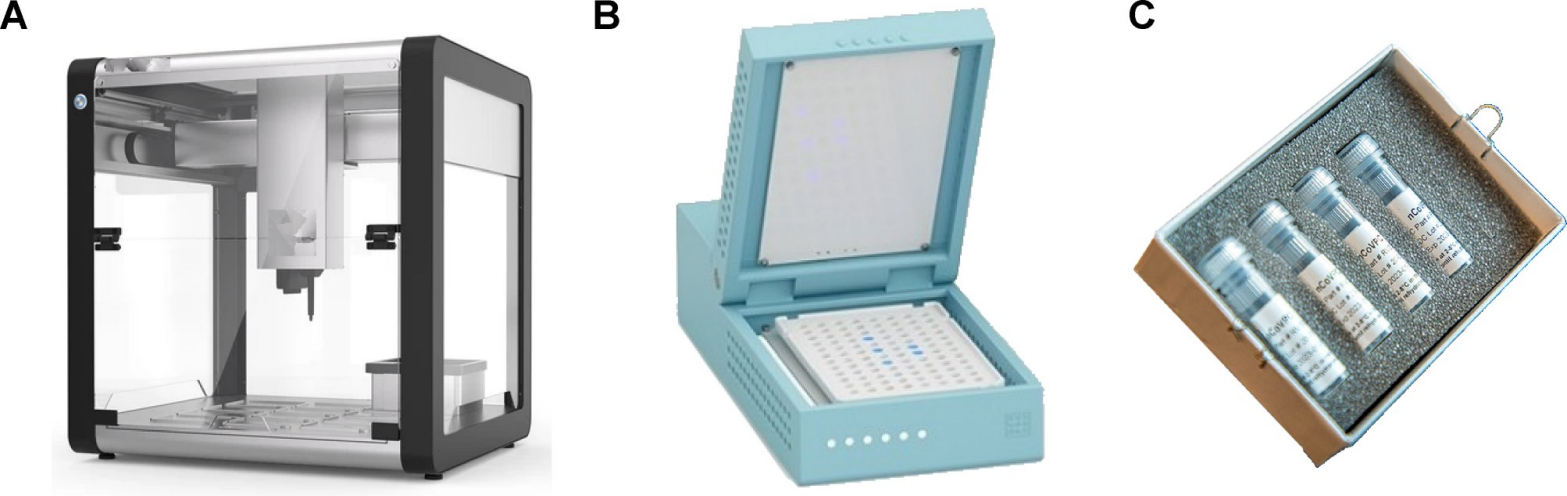
Examples of FOSH designs with potential use in diagnostics. **A**, OpenTrons [28], a liquid handling robot **B**, Miriam, an incubator and reader for isothermal amplifications e.g. LAMP [117]. **C**, US CDC Primer and Probe Kit for diagnostic tests [118].

Community and commercial open source efforts in diagnostic technology to date have focused on four areas: i) open platforms for scaling reactions as exemplified by Opentrons (Fig 3A) [28], an open source lab automation platform that has been working with BP Genomics and the Open Medicine Institute to automate up to 2,400 tests per day and achieve US FDA EUA approval and is now automating COVID-19 testing at the Biomedical Diagnostic Center (CBD) of Hospital Clinic of Barcelona; ii) trying to fill gaps where less attention is being paid by clinical diagnostics companies, such as Chia Bio’s Open qPCR (Fig 3B) environmental test kit for surveillance via surface swabs [111]; iii) distributed reproduction of rapidly-published, lab-scale protocols, seen within the OpenCOVID initiative hosted by Just One Giant Lab [39] which involves many community labs worldwide; iv) initiatives such as the Open Enzyme Collection [93], Free Genes [94] and Biomaker Challenge [112] which are investigating new approaches to foundational technologies such as reagents and instrumentation, with a view to building capacity and resources or global science and medicine to face a future pandemic.

These efforts supplement a wide range of existing open hardware and off-patent, generic biotechnologies which could play a role in expanding molecular testing capabilities [7,113–116].

In addition, a rapidly accumulating list of publications and pre-prints describe protocols that allow bypassing some of the rate limiting steps in the diagnostic protocol [92,119–121]. For example, RNA extraction can be substituted by simply heating the sample to 95°C for 10 minutes [92,121]. Furthermore, the development of alternative diagnostic techniques could help bypass some of the hardware requirements. For example, recently published protocols based on Cas enzymes [106,107,122], ligation-dependent detection [123] and colorimetric RT-LAMP [124,125] obviate the need for a qPCR machine. These protocols require only pipettes and heat blocks or water baths in terms of instrumentation. Readout can then be done via inexpensive lateral flow strips, using a plate reader for which open hardware alternatives exist [29], or even by eye. It is important to note that many of these alternative detection protocols were only shown to work using DNA plasmid controls, or on very small numbers of SARS-CoV-2 patient samples. Therefore, before these are adopted as bona fide diagnostic tests, they need to be thoroughly evaluated, followed by local regulatory approval (see above). Companies are being particularly efficient at this [126], but government-funded labs are also now testing and scaling protocols that are independent of commercial kits and this trend has potential to overcome some of the more acute reagent supply chain issues.

Although most of these enzymes and protocols are patent-locked, they would be available for manufacturing and use in areas where patents were not filed, which likely includes many countries in the Global South. Local manufacturing of health technologies depends on the presence of relevant capacity, infrastructure and regulatory frameworks but is promoted by organisations including the WHO, UNIDO, UNCTAD, UNAIDS, UNICEF and The Global Fund [127]. The implementation of low-cost diagnostic technologies in countries with very limited testing resources could be a game changer in the global control of the COVID-19 pandemic, particularly when combined with protocols for RNA extraction that do not depend on commercial kits ([90–92,119,121], and Table 3), and the generation of reagents that can be distributed and stored at room temperature [128]. For this reason, development of rapid diagnostics for use at the community level was #1 in the WHO’s eight immediate research actions agreed as part of their 2019 Novel Coronavirus Global Research and Innovation Forum [129].

**Table 3:** Diagnostic hardware and reagents with open source alternatives. We have selected FDA/CDC-approved items as an illustration but each country will have its own regulators and agencies with different sets of approved equipment and reagents. Many countries are choosing to simplify or accelerate regulatory approval during the COVID-19 pandemic.

Item	Selected examples of current CDC/FDA-approved items	Status of Open Source alternatives *Note*: *these protocols and devices are not yet nationally approved for diagnostic use in any jurisdiction at the time of writing but they might be approved for use in specific labs and hospitals*.
RNA Extraction Kit	Qiagen QIAamp® Viral RNA Mini Kit Roche MagnaPURE Total Nucleic Acid Kit	There are numerous published protocols to extract viral RNA from swabs using standard laboratory reagents, for example: • Trizol and ethanol technique [90] • Magnetic Bead-based extraction [91] • Direct RT-PCR from crude samples [92]
qRT-PCR Mix	Thermo Fisher Scientific® TaqPath 1-Step RT-qPCR Master Mix	Consists of multiple components: **Enzymes** (e.g. MMLV reverse transcriptase and DNA polymerase) can be produced in most labs from DNA vectors. Early versions are already off-patent and sequences are available from the Open Enzyme Collection [93] and Free Genes [94]. Later versions are patent-protected in some jurisdictions but not all. **dNTPs** require specific manufacturing capabilities and are currently readily available commercially in bulk. **Buffers** typically use standard lab chemicals and are often published but may be proprietary.
Primers	CDC Primer Set and others approved under FDA Emergency Use Authorization [95]	Several primer designs have been published as open data and can be synthesised commercially by any company but for diagnostic use the company and reagent batches should be validated and approved by the relevant local regulatory body or used in a regulated lab.
Positive Control RNA/DNA	Positive controls are distributed with CDC kits by the International Reagent Resource [96]. Genomic RNA positive control available through BEI Resources [97].	SARS-COV-2 genome and control sequences are open data and can be synthesised commercially by any company but must be approved for diagnostic use. Control reagents are available from [98] in the US and from relevant agencies or the WHO elsewhere.
Automated RNA Extraction Platform	Qiagen EZ1 Advanced XL Roche MagNA Pure LC bioMérieux NucliSENS® easyMAG® Instrument	OpenTrons [28] is an open source automated platform that can work with a variety of generic reagents.
qPCR Thermocycler	Applied Biosystems 7500 Fast Dx Real-Time PCR System with SDS version 1.4 software	Open qPCR [99] is partially open hardware. There are currently no fully open alternatives.
Centrifuges	Refrigerated high-speed centrifuge	No open designs yet with suitable specifications. Existing centrifuge designs [100–102] are usually designed for low-speed separation of plasma at ambient temperature. Centrifuges and compatible consumables are widely available from generic manufacturers.
Heat block/Water bath	Heat block/Water bath with 30**–**90°C temperature range	No open heat block designs yet, water baths have been described using microcontrollers and immersion heaters but the simplest available solution is use of a sous vide cooking device [103]. Heat blocks and water baths are widely available from generic manufacturers.
Pipettes	Adjustable micropipettes that meet ISO 8655 Standard in the range 0.5 μl to 1,000 μl	A 3D-printed open source design is available for an adjustable micropipette that meets the ISO 8655 Standard down to 30 μl [104]. Note that this volume is not low enough for use in typical *in vitro* diagnostic testing. Adjustable micropipettes and compatible consumables are widely available from generic manufacturers.

The scientific community is coming together to find and implement faster and cheaper diagnostics, which could be instrumental to control the pandemic, particularly in the medium term amid fears of the so-called “second peak” if and when the strict isolation measures put in place in many countries relax. The diagnostic supply chain issues we face currently highlight the risks of under-investment in public diagnostic infrastructure and of vendor lock-in, resulting in reliance on a small number of suppliers. Efforts from the open hardware community to design and produce rate-limiting equipment and reagents (Table 3, Fig 3) could offer a long-term mitigation strategy, allowing rapid scale up of manufacturing of generic designs and recipes, given sufficient political will and investment.

## Conclusion and outlook

In the sections above, we have summarised a subset of current FOSH related initiatives aiming to meet the global challenge of COVID-19. However, our account is by no means comprehensive, and the situation changes daily. Accordingly it remains important to continuously seek up to date information, for example by following live documents curated by the community on various aspects of the FOSH response [33,37,130], and to join key community portals and mailing lists [23,24,130–132].

For brevity, we omitted discussions of related approaches, for example the introduction and modification of existing tools from veterinary care [133]. We did not discuss the important role open-source software and data-collection initiatives can play in understanding and responding to the ongoing and projected situation [134–136]. Also not covered here are other Open Science tools and projects that could help alleviate many problems in the current scenario [137].

Most of the technology discussed above is classed as a medical device when used in a diagnostic or clinical setting; a challenge faced by only a small number of existing open hardware devices and therefore in need of further research. Moving technology to implementation is perhaps the greatest challenge facing these projects, involving consideration of integration into health care systems, supply chain logistics, regulations, legal liability and political will. The urgency of the current situation has led to a relaxation of regulatory processes by the US Food and Drug Administration [138] and the European Union [139], with some open source projects already reaching formal testing stages e.g. the VentRap ventilator from the Open AIRE forum is ready for evaluation by the Department of Health of the Principality of Asturias in Spain [140].

In conclusion, FOSH approaches are set to play an important part in the global response to our current situation. To what extent they are able to scale a practical response that directly addresses the current wave of need for medical and diagnostic hardware remains to be seen but that is not their only valuable contribution: they are unlocking large and distributed pools of talent, building knowledge, developing innovative designs and fostering capacity and capabilities to tackle this crisis and future emergencies. Like open source software, FOSH works through the eyes and brains of many. The more people join ongoing projects, or launch their own and freely share their progress online, the faster and more effective our united global community will be. The time to join is now.
